# Anisotropic Piezoelectric Properties of Porous (Ba_0.85_Ca_0.15_)(Zr_0.1_Ti_0.9_)O_3_ Ceramics with Oriented Pores through TBA-Based Freeze-Casting Method

**DOI:** 10.3390/ma15113820

**Published:** 2022-05-27

**Authors:** Siyu Ge, Junzhan Zhang, Ying Zhang, Peng Shi, Honghui Wang, Shangyi Liu, Zhifeng Tian, Zongmo Shi

**Affiliations:** 1 College of Materials Science and Engineering, Xi’an University of Architecture and Technology, Xi’an 710055, China; gdayu369@gmail.com (S.G.); xayzhang@xauat.edu (Y.Z.); lsy1261196130@163.com (S.L.); txh1416524133@163.com (Z.T.); shixin2610@163.com (Z.S.); 2 EMRL, Key Laboratory of the Ministry of Education & International Center for Dielectric Research, School of Electronic and Information Engineering, Xi’an Jiaotong University, Xi’an 710049, China; spxjy@mail.xjtu.edu.cn (P.S.); whhdmq87366399@stu.xjtu.edu.cn (H.W.)

**Keywords:** BCZT, freeze casting method, piezoelectricity, oriented porous, anisotropy

## Abstract

Porous (Ba_0.85_Ca_0.15_)(Zr_0.1_Ti_0.9_)O_3_ (BCZT) piezoelectric ceramics with an oriented directional hole structure were prepared by using the tertbutyl alcohol (TBA)-based freeze-casting method. The influences of sintering temperatures on the microstructure and piezoelectric properties of porous BCZT ceramics were investigated both perpendicular and parallel to the freezing direction. With the increase in sintering temperatures and the porosities decreased from 58% to 42%, the compressive strength increased from 14.0 MPa to 25.0 MPa. In addition, the *d*_33_ value of 407 pC/N for the sample sintered at 1400 °C was obtained parallel to the freezing direction, which was 1.40 times that of the other direction.

## 1. Introduction

Pores were introduced into piezoelectric ceramics to provide the properties required for applications, and are widely used in energy harvesters [1], sensors [2], and medical applications [3,4]. Porous piezoelectric ceramics have been a research attraction due to their superior signal-to-noise ratio, electromechanical properties, acoustic impedance, and hydrostatic figure of merit [5]. At present, porous piezoelectric ceramics can be prepared by adding pore-forming agents [6], direct foaming [7], freeze casting [8], and gel casting [9]. The different morphologies of porous ceramics can be obtained, such as foam, honeycomb, and hollow spheres, and the pore sizes can be regulated within a specific range [10]. The freeze-casting method is a new process to manufacture porous piezoelectric ceramics with the advantages of a low cost, low material waste, and simplicity of the preparation process [11].

BCZT ceramics are a lead-free piezoelectric material that has attracted much attention for its high piezoelectric coefficient, which surpasses that of the currently common PZT-based piezoelectric ceramics [12]. To date, the methods of texture [13,14] and doping [15] were adopted for regulating the piezoelectric coefficient and relative permittivity of dense BCZT ceramic materials. Zhang et al. [16] constructed the pore structure of BCZT piezoelectric ceramics in an oriented manner using water-based freeze casting, preparing a lamellar structure with ceramic connections at the edges of the lamellae. The polarization efficiency was significantly enhanced, providing a theoretical basis for increasing the range of applications of the devices. However, for disordered pores, it is more challenging to enhance the quality factor of the device when the relative permittivity was reduced, and the piezoelectric coefficient and mechanical properties are significantly reduced due to the increased porosity. Therefore, it is beneficial to prepare the porous piezoelectric ceramics with oriented pores to modify the dielectric properties. The freezing temperature, solids content, and solvent of the freeze-casting method can be modified to facilitate the preparation of ordered pore structures [17].

In this paper, the comparative experiment investigated the effect of sintering temperatures on the pore structure, dielectric properties, and piezoelectric properties of porous BCZT ceramics. In particular, porous BCZT piezoelectric ceramics parallel to the freezing direction exhibited a lower coercive electric field and higher remnant polarization strength than those perpendicular to the freezing direction. The relationship between the microstructure of oriented pores and the piezoelectric properties of materials was studied and discussed in detail.

## 2. Experimental Section

Barium carbonate (BaCO_3_, 99.7%, Shanghai Macklin Biochemical Co., Shanghai, China), calcium carbonate (CaCO_3_, 99.7%, Sinopharm Chemical Reagent Co., Xi’an, China), titanium dioxide (TiO_2_, 99.7%, Shanghai Macklin Biochemical Co., Shanghai, China), and zirconium dioxide (ZrO_2_, 99.7%, Sinopharm Chemical Reagent Co., Xi’an, China) were weighed according to the nominal composition of (Ba_0.85_Ca_0.15_)(Zr_0.1_Ti_0.9_)O_3_ and wet-ball-milled for 10 h using ZrO_2_ balls and an ethanol medium. The wet ball mill uses a ball mill jar made of polyurethane with a rotation speed of 300 r/min. After mixing and drying, the powder was calcined at 1200 °C for 2 h.

**Figure 1:**
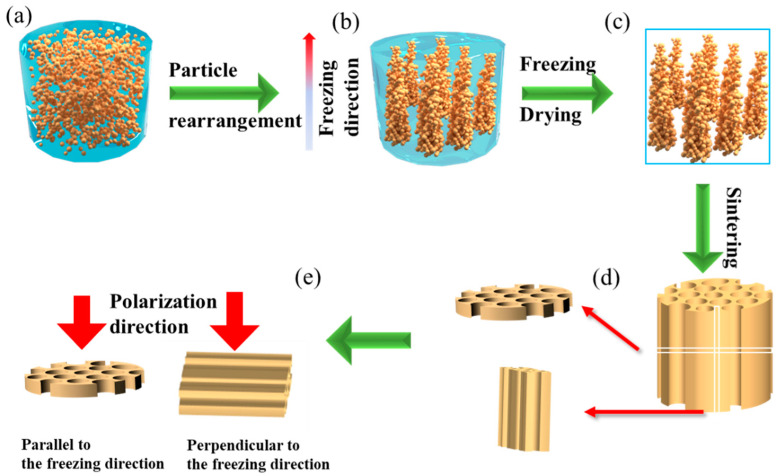
Schematic diagram of porous BCZT piezoelectric ceramics prepared by freeze casting. (**a**) slurry preparation, (**b**) slurry freezing solidification and particle rearrangement, (**c**) sublimation drying gives porous ceramic blanks, (**d**) sintering and cutting samples, (**e**) the samples with a direction of polarization parallel or perpendicular to the freezing direction are defined as parallel samples or perpendicular samples.

Then, 1.2 wt% acacia (99.5%, Sinopharm Chemical Reagent Co., Xi’an, China) and 1.5 wt% polyvinyl butyral (PVB, 99.5%, Sinopharm Chemical Reagent Co., Xi’an, China) were dissolved into a mixed solvent of tertbutyl alcohol (TBA, 99.5%, Sinopharm Chemical Reagent Co., Xi’an, China) and deionized water to obtain the precursor solution as shown in Figure 1a. Then, the solution was mixed with (Ba_0.85_Ca_0.15_)(Zr_0.1_Ti_0.9_)O_3_ powders and ball-milled for 6 h. The well-dispersed slurry suspension was poured into a nylon mold with a copper bottom for unidirectional solidification as shown in Figure 1b, and placed in a freeze dryer for sublimation, leaving oriented pore channels with TBA crystals as templates to obtain dried porous ceramic blanks as shown in Figure 1c. Afterward, the samples were sintered in a muffle furnace at 1300–1450 °C for 3 h and cooled with the furnace. The samples were cut crosswise and longitudinally into Φ10 mm × 1 mm plate as shown in Figure 1d. Samples with polarization directions parallel or perpendicular to the freezing direction were defined as samples parallel to the freezing direction, and samples perpendicular to the freezing direction are shown in Figure 1e.

The phase compositions of the samples were characterized using X-ray diffraction (XRD, PANalytical Empyrean, PANalytical, Netherlands) with Cu Kα radiation (5° ≤ 2*θ* ≤ 90°) under the following measurement conditions: wavelength λ = 1.54 Å, tube voltage of 40 kV, tube current of 40 mA, scanning step of 0.02°, scanning speed of 5°/min. Tungsten wire scanning electron microscope (SEM, TESCAN VEGA3, Brno, The Czech Republic) with acceleration voltage of 20 kV was used to analyze the micromorphologies of the samples. The statistical distributions of pore sizes were measured using the Image-Pro Plus software. The porosity and density of the samples were obtained according to the Archimedes drainage method, where the immersion liquid for the density measurements was water. A universal testing machine (Jinan Tianchen Testing Machine Manufacturing Co., Ltd. Jinan, China) was used to test compressive strength. For the dielectric and piezoelectric properties test, the silver electrodes were formed by screen printing a thin layer of silver paste onto both surfaces of each sample, followed by heat treatment at 600 °C for 20 min to form silver electrodes. The samples were polarized in a silicone oil bath at room temperature under an electric field of 1 kV/mm, which was imposed for 30 min, and then the samples were aged for 24 h. Relative permittivity of the samples was measured using an impedance analyzer (Keysight E4990A, Santa Clara, CA, USA). Determination of dielectric behavior was measured with a precision LCR meter (Agilent E4980A, Santa Clara, CA, USA) and temperature controller (Agilent HP 4284ALRC) at a frequency of 1 kHz by 3 °C/min over a temperature range of 25 °C to 180 °C, where the oscillation voltage was 1 V. The Agilent HP 4284ALRC regulates temperature via PID numerical control. Ferroelectric hysteresis loops (*P*-*E*) were measured at room temperature by a ferroelectric tester (TF analyzer 2000, aixACCT Systems, Aachen, Germany) at 1 Hz. Piezoelectric properties of BCZT ceramics were measured by *d*_33_ piezoelectric instrument (ZJ-6A, Institute of Acoustics, Academia Sinica, Beijing, China).

## 3. Results and Discussion

**Figure 2:**
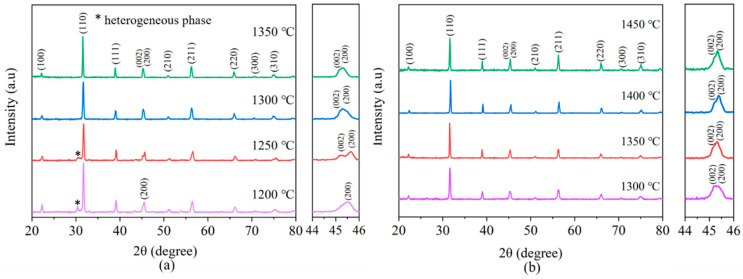
(**a**) XRD patterns of BCZT powders calcined at the different temperatures with the enlarged views of the peaks 2*θ* ≈ 44–46°. (**b**) XRD patterns of porous BCZT piezoelectric ceramics sintered at different temperatures with the enlarged views of the peaks 2*θ* ≈ 44–46°.

Figure 2a shows the XRD results of calcined BCZT powders at different temperatures (1200–1350 °C). At a calcination temperature of 1200 °C, a BT-based solid solution with a cubic phase structure begins to form, although a small amount of the heterogeneous phase was present. A comparison with the XRD standard PDF card shows that the heterogeneous phase was associated with Ba_3_Ca_2_Ti_2_O_9_ (JCPDS: 042-0535). When calcined at 1250 °C, the heterogeneous phase still appears and the peaks in the 2*θ* range of 44–46° split into (002) and (200) peaks. A comparison with the P4 mm space group of BaTiO_3_ in the conventional tetragonal phase (JCPDS: 05-0626) indicates that the synthesized powder was in a tetragonal phase structure [18]. With the increase in calcination temperature from 1300 °C to 1350 °C, the phase structure of the synthesized BCZT powder did not change. Figure 2b illustrates the XRD results of the porous BCZT piezoelectric ceramics sintered at different temperatures. At a sintering temperature of 1300 °C, the (200) peak of the porous BCZT piezoelectric ceramic splits into (002) and (200) peaks in the 2*θ* range of 44–46°, corresponding to the diffusion of Ca^2+^ (A-site) and Zr^4+^ (B-site) ions into Ba^2+^ (A-site) and Ti^4+^ (B-site) [12,19]. The formation of the tetragonal phase was also confirmed by comparison with JCPDS card number 05-0626, which does not form a heterogeneous phase. With the sintering temperature continuing to increase to 1450 °C, no heterogeneous phases were present and the (200) peak remained split into the (002) and (200) peaks in the 2*θ* range of 44–46°, confirming a tetragonal phase structure. As a consequence, different sintering temperatures do not cause significant changes in the phase structure of porous BCZT piezoelectric ceramics [20]. The increasing intensity of the diffraction peaks and sharpness indicated the increasing crystallinity of the samples with increasing sintering temperatures.

**Figure 3:**
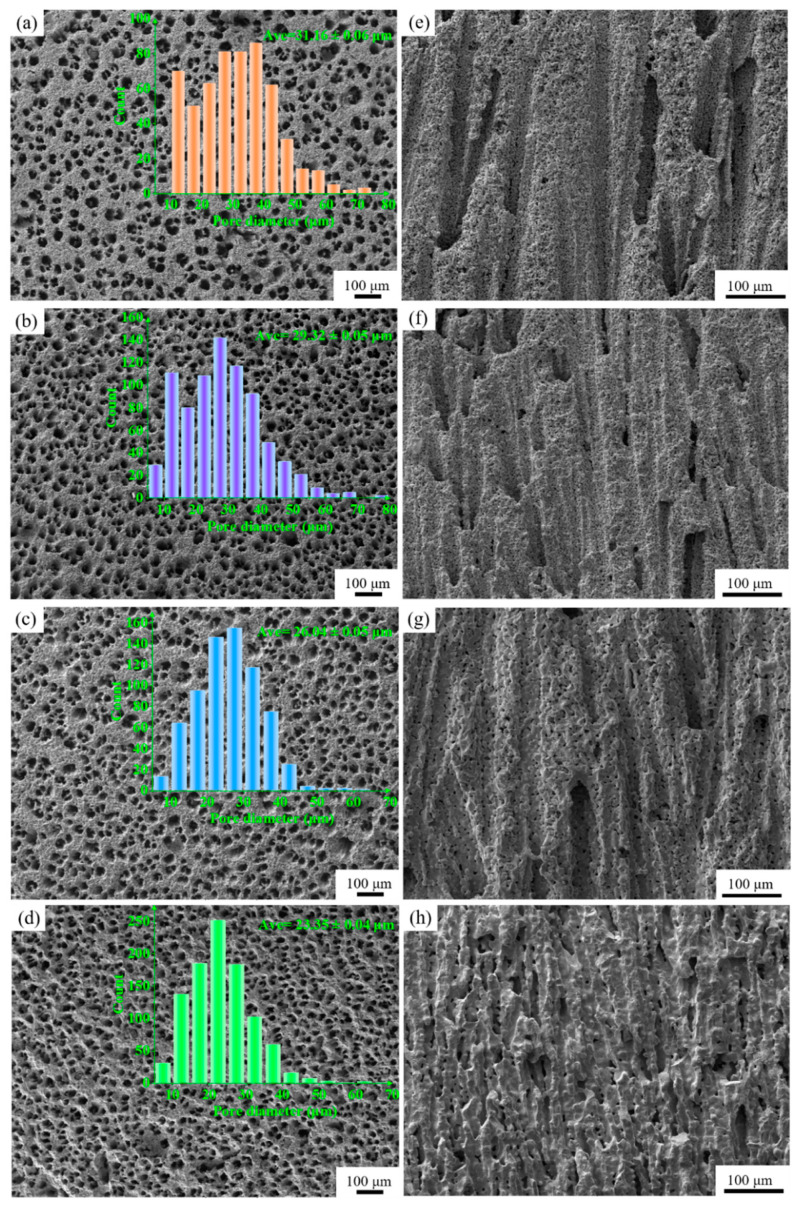
The typical SEM micrographs of cross-section in the porous BCZT ceramics sintered at different temperatures: (**a**) 1300 °C, (**b**) 1350 °C, (**c**) 1400 °C, (**d**) 1450 °C. (Insets are the pore size distribution.) The microstructures of longitudinal section in the porous BCZT samples at different sintering temperatures: (**e**) 1300 °C, (**f**) 1350 °C, (**g**) 1400 °C, (**h**) 1450 °C.

Figure 3a–d presents the cross-section SEM images of BCZT porous ceramics. As can be seen from the micrographs, there was a relationship between hole size and sintering temperature. With increasing sintering temperatures, the average pore sizes reduced from 31.16 µm to 23.35 µm, whereas the number of pores gradually increased. Figure 3e–h indicates longitudinal section SEM images of BCZT porous ceramics. All samples showed straight and long pore structures, indicating that the pore channels were aligned in the freezing direction. Porous structures were extremely anisotropic, and the ceramic had tubular-shaped structures with a large length-to-diameter ratio. It was shown that the porous BCZT ceramics obtained by the freeze-casting method have a distinct honeycomb structure and one-dimensionally ordered pore channels.

**Figure 4:**
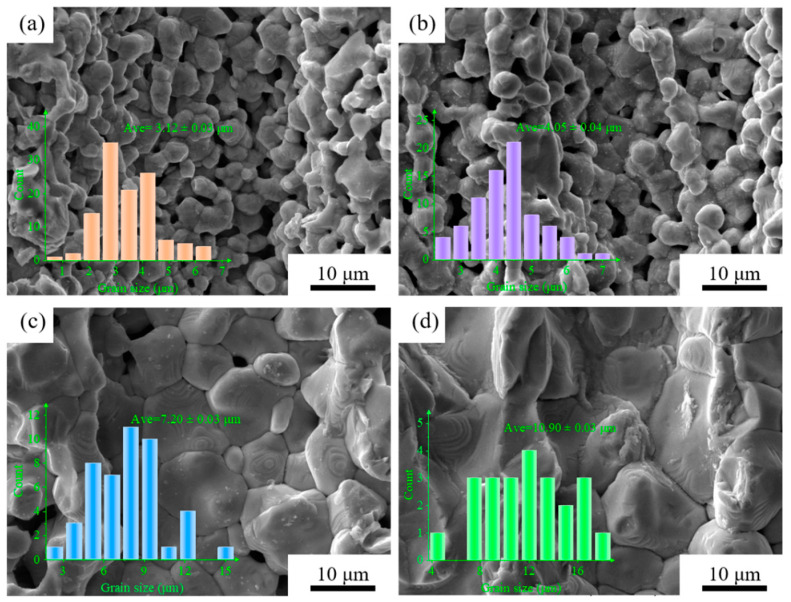
Pore walls of porous BCZT piezoelectric ceramics at different sintering temperatures: (**a**) 1300 °C, (**b**) 1350 °C, (**c**) 1400 °C, (**d**) 1450 °C.

Figure 4 shows the pore walls of the porous BCZT piezoelectric ceramics at different sintering temperatures. As the sintering temperature increased, the grains grew progressively, and the pore walls became denser. Below 1350 °C, the grain particles were not sufficiently interconnected, and there were many small pores in the pore walls. However, the grains were large and tightly connected, with no small pores in the pore walls at 1450 °C. There were no clearly interconnected pores in the ceramics wall. In addition, the pore channels were aligned with the direction of the electro-polarization field, which may affect the polarization process, as will be discussed later.

**Figure 5:**
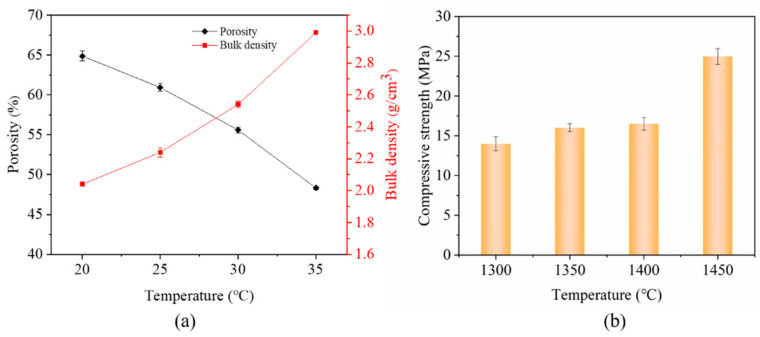
(**a**) Apparent porosity and bulk density of porous BCZT piezoelectric ceramics at different sintering temperatures, (**b**) compressive strength of porous BCZT piezoelectric ceramics at different sintering temperatures.

Figure 5a shows the apparent porosities and bulk densities of porous BCZT piezoelectric ceramics sintered at different temperatures. It can be seen that the apparent porosity gradually diminished as the sintering temperature increased, whereas the bulk density progressively increased accordingly. When the sintering temperature was 1450 °C, the porosity was at a minimum at 42%, while the bulk density was at a maximum at 3.29 g/cm^3^. This may be attributed to the structure of the pore walls tending to be as dense as the stacking density, as well as the tight connection of the ceramic grain during sintering to fill the pore areas. Figure 5b shows the compressive strength of porous BCZT piezoelectric ceramics. A large macroscopic compressive strength was obtained due to the particles stacking much more tightly when the sintering temperature was increased; the maximum compressive strength was 25.0 MPa. However, when the sintering temperature was 1450 °C, the sample became more glassy, resulting in a reduction in porosity and partial destruction of the pore structure.

**Figure 6:**
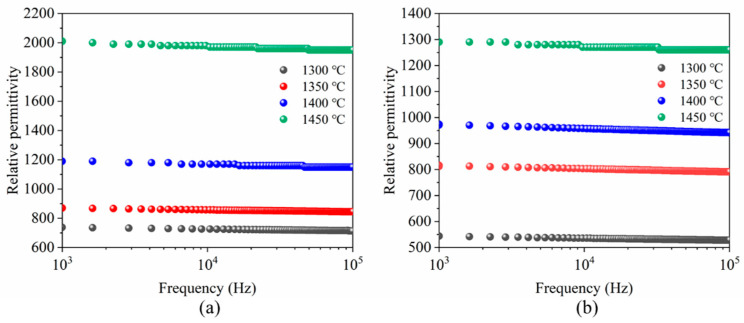
Relative permittivity of porous BCZT piezoelectric ceramics sintered at different temperatures (**a**) parallel to the freezing direction, (**b**) perpendicular to the freezing direction.

Figure 6a shows the relative permittivity of porous BCZT piezoelectric ceramics at different sintering temperatures in both directions. It was observed that the relative permittivity of porous BCZT piezoelectric ceramics increased in both directions as the sintering temperature increased. As the sintering temperature increased, the porosity decreased and the grain size increased, resulting in an increase in bulk density. The thickness of the insulating grain boundary layer (relatively large compared to the thickness of the grain) decreased and, therefore, *ε*_r_ increased with an increasing sintering temperature [21]. At the same time, it can be seen that the relative permittivity of porous BCZT ceramics parallel to the freezing direction was higher than those perpendicular to the freezing direction at the same sintering temperature. For porous BCZT piezoelectric ceramics (parallel to the freezing direction), the pore channels were parallel to the polarization direction, correspondingly causing an increase in the volume fraction of the BCZT phase along the polarization direction, increasing the polarization efficiency [22]. This, in turn, led to a corresponding increase in the relative permittivity, which was in agreement with the results of Bowen C R [23].

**Figure 7:**
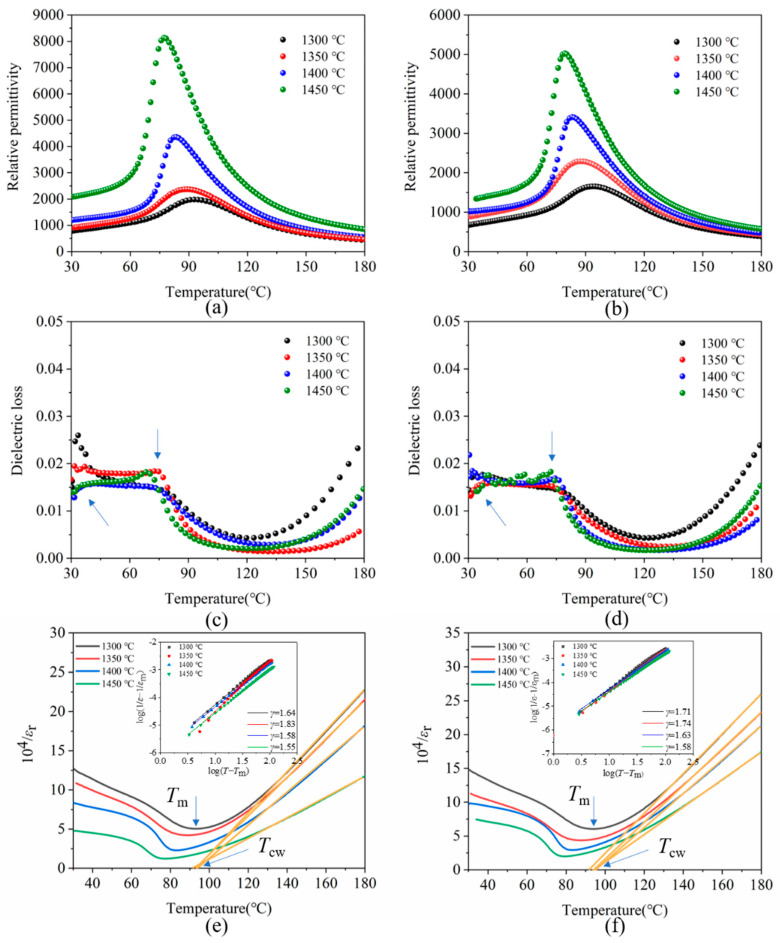
(**a**) Relative permittivity, (**c**) dielectric loss, and (**e**) inverse relative permittivity as a function of measuring temperature and sintering temperature of porous BCZT piezoelectric ceramics sample (parallel to the freezing direction). (**b**) Relative permittivity, (**d**) dielectric loss, and (**f**) inverse relative permittivity as a function of measuring temperature and sintering temperature of porous BCZT piezoelectric ceramics sample (perpendicular to the freezing direction). (Insets are the corresponding curves of log(1/*ε* − 1/(*ε*_r_)_m_) against log(*T* − *T*_m_).)

Figure 7a–d shows the temperature-dependent relative permittivity and dielectric loss of porous BCZT piezoelectric ceramics with different sintering temperatures at 1 kHz (Figure 7a,c for those parallel to the freezing direction; Figure 7b,d for those perpendicular to the freezing direction). When the sintering temperature increased, the relative permittivity tended to increase for both directions. The relative permittivity peak at *T*_m_ (Curie temperature) was wider (diffusive behavior) for samples sintered at 1300 °C and got steeper as the sintering temperature increased (to 1450 °C). With the sintering temperature increased from 1300 °C to 1450 °C, the *T*_m_ shifts towards lower temperatures (from 94 °C to 77 °C with those parallel to the freezing direction; from 94 °C to 79 °C with those perpendicular to the freezing direction). This phenomenon was consistent with the reported results for Ca^2+^ -doped BaTiO_3_ ceramics [24], which was closely related to the substitution of A for Ba^2+^ by Ca^2+^. It was shown that Ba^2+^ (1.61 Å) and Ca^2+^ (1.34 Å) enter the A-site of the ABO_3_ chalcogenide structure, whereas Zr^4+^ (0.72 Å) and Ti^4+^ (0.605 Å) occupy the B-site [25]. In addition, it was consistent with the experimental results of Li et al. [26]. The shift of *T*_m_ towards a lower temperature with the increase in sintering temperature may be attributed to the cell volume effect induced by a larger grain size [26,27]. Two peaks were observed in the dielectric loss versus temperature plot. It was the phase transition between the rhombohedral and tetragonal phases that was depicted by the anomaly near room temperature. Near 80 °C was the transition from the tetragonal phase to the cubic phase, causing a change in dielectric loss. The dielectric loss gradually increased at higher temperatures, whose higher values indicated space charge polarization and the associated ionic conductivity [28]. Nevertheless, the maximum value of dielectric loss remains <0.03.

Further confirmation of the ferroelectric diffusivity of the phase transition of porous BCZT ceramics sintered at different temperatures was quantitatively characterized.

(1) $$\frac{1}{\varepsilon_{r}}=\frac{T-T_{\mathrm{cw}}}{C}\;(T\;>\;T_{\mathrm{cw}})$$

where *ε*_r_ is the relative permittivity, *T*_cw_ is the Curie–Weiss temperature and *C* is the Curie–Weiss constant. Figure 7e,f summarizes the results of the Curie–Weiss law fit. The relative permittivity of the sample at 1300 °C was in accordance with the Curie–Weiss law (Equation (1)). For the remaining samples, the relative permittivity of the sintered samples deviated from the Curie–Weiss law above the Curie temperature as the sintering temperature increased. The dielectric behavior of complex ferroelectric materials was explained in terms of diffuse phase transition (DPT) by using a modified empirical formula (Equation (2)) proposed by K. Uchino [29].

(2) $$\frac{1}{\varepsilon_{r}}-\frac{1}{{\left(\varepsilon_{r}\right)}_{m}}=\frac{{\left(T-T_{m}\right)}^{\gamma }}{C}$$

where *T*_m_ is the temperature (corresponding to *ε*_r_(max)) at which the *ε*_r_ value reaches the maximum, *γ* and *C* are all constants, and the γ value is between 1 and 2. The limit values *γ* = 1 and *γ* = 2 correspond to the Curie–Weiss law equations, which characterize normal and ideal relaxation ferroelectrics, respectively [29]. For the sample sintered at 1350 °C (parallel to the freezing direction), values of *γ* were as high as 1.83, indicating the typical relaxation behavior. Along with the increase in sintering temperature, the value of *γ* increased and then decreased. The diffuse phase transition behavior observed in the samples sintered at 1350 °C can be assigned to the refinement of the ferroelectric domain and weakening of long-range ferroelectric interactions [30,31].

**Figure 8:**
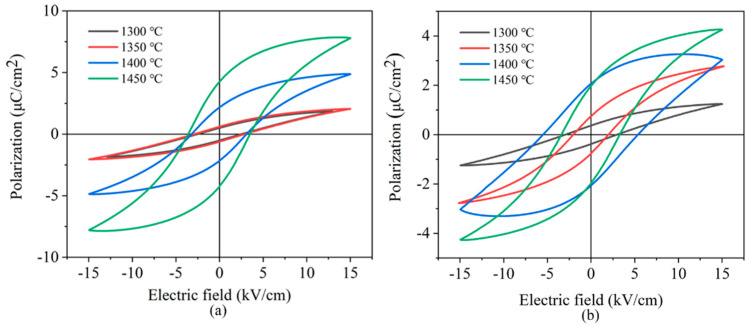
Ferroelectric hysteresis loops of porous BCZT piezoelectric ceramics sintered at different temperatures (**a**) parallel to the freezing direction, (**b**) perpendicular to the freezing direction.

Figure 8 shows the *P*–*E* loops of porous BCZT ceramics. The samples showed symmetrically shaped hysteresis loops, demonstrating that all porous BCZT ceramics (parallel and perpendicular to the freezing direction) showed a good ferroelectric response. Table 1 shows the coercive electric field and remnant polarization intensity in relation to porous BCZT (parallel to the freezing direction), with *P*_r_ increasing from 0.50 μC/cm^2^ to 4.24 μC/cm^2^ and *E*_c_ increasing from 2.52 kV/cm to 3.57 kV/cm as the sintering temperature increased from 1300 °C to 1450 °C. The passive porous phase (AIR) in porous BCZT piezoelectric ceramics was reduced and the pore walls became dense, promoting an increase in polarization efficiency and, consequently, an increase in the remnant polarization intensity. From the literature, as the sintering temperature approached, the grain size and domain size expanded, requiring a significantly high electric field to switch the domains [32]. As the sintering temperature increased from 1300 °C to 1450 °C, the *P*_r_ of porous BCZT ceramics (perpendicular to the freezing direction) increased to 2.06 μC/cm^2^ and then decreased, as shown in Table 1. Porous BCZT ceramics (parallel to the freezing direction) exhibited a relatively small coercive electric field and high remnant polarization strength at 1400 °C compared to those perpendicular to the freezing direction. Samples perpendicular to the direction of freezing were polarized in a direction perpendicular to the aperture. Meanwhile, R Khachaturyan demonstrated that elongated pores aligned perpendicular to the applied field exhibited a broadening of the ferroelectric hysteresis loops [33]. However, the least disruption to the electric field was observed when elongated pores were aligned parallel to the electric field [34]. The amount of the liquid phase increased at 1450 °C for porous BCZT ceramics, causing a slow transformation of part of the oriented pore structure into a randomly distributed pore structure, which consequently enlarged the unpolarized region of the porous material and degraded the polarization efficiency [35].

**Figure 9:**
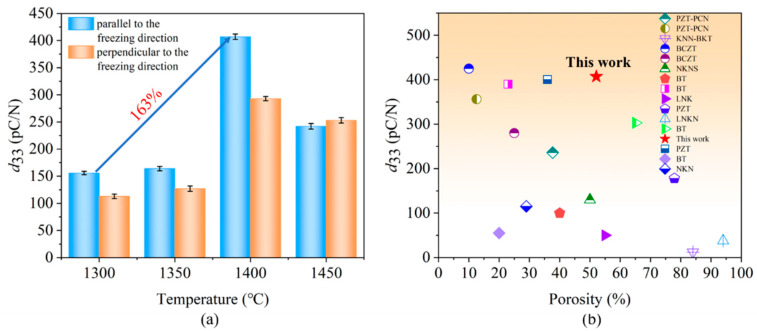
(**a**) Piezoelectric coefficient of porous BCZT piezoelectric ceramics sintered at different temperatures (parallel and perpendicular to the freezing direction), (**b**) comparison of the *d*_33_ of porous BCZT piezoelectric ceramics with other porous piezoelectric materials. The other porous piezoelectric materials include LNK (porous lithium sodium potassium niobate ceramics) [7], NKN (porous (Na_0.5_K_0.5_)NbO_3_ ceramics) [22], PZT (porous lead zirconate titanate ceramics) [37,39], BT (porous BaTiO_3_ ceramics) [36,44,45,46], LNKN (porous lithium sodium potassium niobate ceramics) [39], NKNS (porous (Na_0.52_K_0.48_)(Nb_0.95_Sb_0.05_)O_3_ ceramics) [47], KNN-BKT (porous (1-x) (K, Na)NbO_3_. x (Bi, K)TiO_3_ ceramics) [48], BCZT (porous 0.5Ba(Ca_0.8_Zr_0.2_)O_3_-0.5(Ba_0.7_Ca_0.3_)TiO_3_ ceramics) [38], and PZT-PCN (porous lead zirconate titanate-lead cobalt niobate 0.8Pb(Zr_1/2_Ti_1/2_)O_3_-0.2Pb(Co_1/3_Nb_2/3_)O_3_ ceramics) [49].

The longitudinal piezoelectric coefficient (*d*_33_) of porous BCZT piezoelectric ceramics (in both parallel and perpendicular to the freezing direction) are shown in Figure 9a. With the increase in sintering temperatures, the longitudinal piezoelectric coefficient initially increased and subsequently decreased. The maximum value of *d*_33_ of 407 pC/N appeared at 1400 °C and increased by 163% for the sample (parallel to the freezing direction) sintered at 1300 °C. Moreover, the *d*_33_ of porous BCZT ceramics parallel to the freezing direction was comparatively high compared to porous BCZT ceramics perpendicular to the freezing direction. Under the same sintering temperature of 1400 °C, the *d*_33_ was 407 pC/N for the sample parallel to the freezing direction, which was 28% larger than that of the sample perpendicular to the freezing direction. The low porosity and high bulk density of the oriented through-pore structure formed at the sintering temperature of 1400 °C is attributed to the fact that the pore walls become more dense and thus increase the polarization efficiency. Furthermore, as the polarization direction is parallel to the freezing direction, a higher degree of hole alignment may make the domain walls with small areas rotate easily and therefore increase the piezoelectric properties [36]. Figure 9b shows a comparison of *d*_33_ with other porous piezoelectric materials, particularly lead-free porous piezoelectric ceramics. In this work, the sample sintered at 1400 °C exhibited a high *d*_33_ value of 407 pC/N accompanied by a porosity of 52%. Further comparison with the water-based porous PZT sample [37] revealed that the piezoelectric properties of the porous BCZT sample (parallel to the freezing direction) were superior for the same porosity. The top *d*_33_ of porous BCZT piezoelectric ceramics (parallel to the freezing direction) is close to the *d*_33_ of much denser BCZT in Ref. [38]. The large surface area due to the special honeycomb pore structure increases the surface tension of the material and thus reduces the domain size to some extent. A small domain structure in porous piezoelectric ceramics facilitates the piezoelectric properties. Combined with theoretical and experimental results on porous PZT ceramics [39,40,41,42], having a ceramic matrix with unidirectional pores plays a key role in enhancing the piezoelectric properties. The *d*_33_ of the BCZT single crystal was 200 pC/N [43], whereas the dense BCZT ceramics *d*_33_ was 620 pC/N [12]. It is also worth noting that porous materials have poor piezoelectric properties at a high porosity, which may be attributed to factors such as pore size, pore size distribution, or the type of pore structure. The oriented pore structure BCZT ceramics in this study have superior piezoelectric properties while retaining a high porosity. A more in-depth study of oriented pore structure ceramics prepared by TBA-based freeze casting could enhance piezoelectric properties.

## 4. Conclusions

The oriented porous BCZT piezoelectric ceramics with a high porosity of 42–58% were successfully prepared using the TBA-based freeze casting method. The mechanical and electrical properties of polarized porous ceramics parallel and perpendicular to the freezing direction were investigated. Long, straight-oriented pore structures were formed and the pore sizes decreased from 31.16 μm to 23.35 μm. The compressive strength increased from 14.0 MPa to 25.0 MPa with increasing sintering temperatures. The Curie temperature of porous piezoceramics (parallel to the freezing direction) decreased from 94 °C to 77 °C, whereas the dielectric loss increased from 0.0085 to 0.012 accompanied by the reduction in Curie temperature. The talented piezoelectric coefficient of 407 pC/N for the sample with 52% porosity parallel to the freezing direction was achieved with *P*_r_ = 2.17 μC/cm^2^ and *ε*_r_ = 974. This work shows that the TBA-based freeze-casting method not only compensated for the reduction in the *d*_33_ value due to the increased porosities, but was also applied to piezoelectric ceramics with anisotropic properties.

## Figures and Tables

**Table 1:** Effect of sintering temperature on the coercivity field and remnant polarization intensity for the porous BCZT piezoelectric ceramics.

		1300 °C	1350 °C	1400 °C	1450 °C
(parallel to the freezing direction)	*E*_c_ (kV/cm)	2.52	2.84	3.21	3.57
	*P*_r_ (μC/cm^2^)	0.50	0.60	2.17	4.24
(perpendicular to the freezing direction)	*E*_c_ (kV/cm)	2.84	1.98	5.49	3.39
	*P*_r_ (μC/cm^2^)	0.37	0.74	2.06	1.97

## Data Availability

The data presented in this study are available on request from the corresponding author.
